# Novel characteristics of the temporal transition to maximum tongue pressure in Parkinson’s disease: A pilot study

**DOI:** 10.1016/j.prdoa.2024.100244

**Published:** 2024-02-25

**Authors:** Sachi Hayasaka, Kozo Hatori, Shuko Nojiri, Taku Hatano, Takao Urabe, Akito Hayashi, Nobutaka Hattori, Toshiyuki Fujiwara

**Affiliations:** a Department of Rehabilitation Medicine, Juntendo University Urayasu Hospital, Tomioka 2-1-1, Urayasu-shi, Chiba 279-0021, Japan; b Department of Rehabilitation Medicine, Juntendo University Graduate School, Hongo 3-1-3, Bunkyo-ku, Tokyo 113-8431, Japan; c Medical Technology Innovation Center, Juntendo University, Hongo 3-1-3, Bunkyo-ku, Tokyo 113-8431, Japan; d Department of Neurology, Juntendo University Faculty of Medicine, Hongo 3-1-3, Bunkyo-ku, Tokyo 113-8431, Japan; e Department of Neurology, Juntendo University Urayasu Hospital, Hongo 3-1-3, Bunkyo-ku, Tokyo 113-8431, Japan

**Keywords:** Repeated measurements, Isometric tongue pressure, Temporal transition, Bradykinesia, Parkinson’s disease

## Abstract

**Introduction:**

The reason why maximum tongue pressure (MTP) decreases in patients with Parkinson’s disease (PD) remains unclear. Repeated measurements of isometric force and MTP may be useful for analyzing muscle wasting and force generation. The purpose of this pilot study was to evaluate the clinical characteristics and temporal transition of MTP in PD and normal control (NC) groups.

**Methods:**

There were 18 participants in this study: 10 with PD and 8 NCs. The MTP was measured 20 times at regular intervals. The area under the curve of MTP temporal transitions, time to reach MTP, and total transition time of the tongue pressure (time to return to baseline) were compared between the groups.

**Results:**

MTP decreased from baseline in PD subjects. Unlike NCs, PD subjects showed diverse and inconsistent temporal transitions. The decrease in MTP and delays in time to reach MTP and time to return to baseline were significantly greater in PD subjects (p < 0.05), while there was no group difference in area under the curve values. According to repeated-measures ANOVA, MTP was not different over time between PD subjects and NCs.

**Conclusion:**

In this study, muscle fatigue did not affect the decrease in MTP seen in PD subjects, or the diversity and inconsistency of the temporal transition in MTP in that group. These findings indicate that the motor control needed for the repeated, identical movements associated with MTP generation may be impaired in PD patients.

## Highlights

• Repeated measurements of tongue isometric force allowed assessment of the extent of muscle wasting and temporal transitions of force generation.
• The decrease in maximal tongue pressure seen in Parkinson’s disease subjects was not attributable to peripheral fatigue.
• The motor control needed for the repeated, identical movements associated with maximum tongue pressure generation was impaired in Parkinson’s disease subjects.

## 1 Introduction

Maximum tongue pressure (MTP) is involved in all stages of swallowing, which is a complex process involving voluntary and reflexive movements [1]. The MTP was reported to be decreased in Parkinson’s disease (PD) patients compared with that in age-matched healthy controls [2]. Furthermore, tongue weakness and the associated prolongation of pressure generation may be related to bradykinesia, and are cardinal features of the basal ganglia dysfunction seen in PD [3]. Although dysphagia may manifest in the early stage of PD [4], early clinical identification of latent swallowing disorders is generally quite difficult.

Few studies have investigated the relationship between tongue pressure generation processes, including MTP, and major motor symptoms (such as bradykinesia) seen in PD [5]. Investigation of dysphasia caused by tongue bradykinesia is needed. To clinically evaluate the pathophysiology of muscle weakness (including tongue pressure) in PD patients, temporal transitions and isometric force generation should be assessed. The MTP was investigated in this study via repeated measurements of tongue isometric force. This method for evaluating maximum muscle force is not affected by joint movement or inertial force. Moreover, repeated measurements allowed assessment of the extent of muscle wasting and temporal transitions of force generation. In a previous study, subclinical aspiration was observed in > 30 % of healthy elderly subjects, and the aspiration group had significantly lower isometric maximum anterior and posterior tongue pressures than the non-aspirated group [6].

On the basis of the above findings, to analyze tongue bradykinesia in PD, we performed repeated measurements of the MTP in PD subjects and normal controls (NCs).

## 2 Methods

### 2.1 Participants

Eighteen participants (ten PD subjects and eight NCs) provided written informed consent to participate in this study from 2019 to 2021. This study was approved by the Institutional Review Board of Juntendo University Urayasu Hospital. All PD cases were sporadic, and the diagnosis was based on the criteria of the International Parkinson’s Movement Disorders Society [7]. Patients with consciousness disturbance, cognitive decline precluding testing, higher cerebral dysfunction, inability to follow instructions, and dysphagia (e.g., cancer of the tongue and pharynx) were excluded from this study. PD patients undergoing deep brain stimulation or levodopa-carbidopa continuous gel infusion therapy were also excluded.

### 2.2 Study design

MTP measurements were performed during the “on” state in all PD subjects (i.e., while taking anti-PD medications). The UPDRS and HY stage examinations were also performed during the “on” stage (Table 1). MTP was measured 20 times at 10-second intervals using an isometric tongue pressure measuring device (TPM-02; JMS, Hiroshima, Japan); the maximum value for each measurement was taken as the MTP. This method, in which the anterior half of the tongue is pressed against the hard palate with maximum force, is widely used in clinical practice in Japan because of its simplicity, reliability, and practicality [8]. The Iowa Oral Performance Instrument (IOPI Medical, Woodinville, WA, USA) is used worldwide for tongue pressure measurements, but its use in Japan has not been legalized. MTPs obtained with this device are comparable with those obtained using the Iowa Oral Performance Instrument [8]. Prior to the study, our subjects completed three trial exercises to obtain a stable tongue pressure value.

**Table 1:** Demographic and clinical characteristics of both groups.

	PD subjects (n = 10)	NCs (n = 8)	
	Mean ± SD	Mean ± SD	p-value
Age [y]	66.5 ± 7.7	59.4 ± 8.3	0.08
Gender [female/male]	4 / 6	3 / 5	0.53
Disease duration [y]	11.5 ± 7.3	( −)	( −)
MDS-UPDRS Part III score	45.2 ± 19.1	( −)	( −)
H&Y stage	3.2 ± 0.9	( −)	( −)
MTP [kPa]	32.0 ± 6.7	42.0 ± 7.8	0.01
AUC [kpixel]	105.7 ± 27.7	97.2 ± 31.3	0.49
Ti_Tm [s]	1.38 ± 0.90	0.52 ± 0.39	0.00
TTT [s]	2.25 ± 0.94	1.11 ± 0.58	0.00

PD, Parkinson’s disease; NC, normal control; H&Y stage, Hoehn and Yahr stage; MDS-UPDRS, Unified Parkinson Disease Rating Scale; MTP, maximum tongue pressure; AUC, area under the curve of the MTP temporal transition; Ti_Tm, time from the start of tongue pressure generation (Ti) to MTP; TTT, total transition time of the tongue pressure (time to return to baseline); y, years; s, second.

In all subjects, 20 MTP temporal transitions were recorded and grouped (MTPs 1–5, 6–10, 11–15, and 16–20). Rather than providing all MTPs, the general patterns of their temporal transitions are presented for both groups.

The tongue pressure measuring device used in this study monitors tongue pressure every 0.05 s. The time from the start of tongue pressure generation to MTP was defined as Ti_Tm, and the total transition time of the tongue pressure (time to return to baseline) was defined as TTT. The area under the curve (AUC) of each MTP temporal transition was compared between groups (tongue pressure in the anterior half of the tongue). The temporal transition of each MTP was also converted into an image analyzed with ImageJ (ver. 1.47; NIH, Bethesda, MD. USA). To analyze the distribution of MTP according to TTT, a scatterplot for MTP and TTT, and density curves of the former, were generated for both groups. The MTP analysis parameters and temporal transitions are shown in Appendix A, along with schematic diagrams.

### 2.3 Statistical analysis

All statistical analyses were performed using SAS 9.4 software (SAS Institute, Cary, NC, USA), with p < 0.05 considered significant. Descriptive statistics for MTP, AUC, Ti_Tm, and TTT were generated for each group. Student’s *t*-test was used to compare ordinal and continuous variables. Mixed-effects ANOVA of MTP was performed with group as the between-subjects effect and force amplitude as a covariate. In the one-way repeated-measures ANOVA of both groups, the within-subjects factor was MTP.

## 3 Results

Demographic and clinical data are reported in Table 1.

### 3.1 Temporal transition and distribution of MTPs

**Fig. 1:**
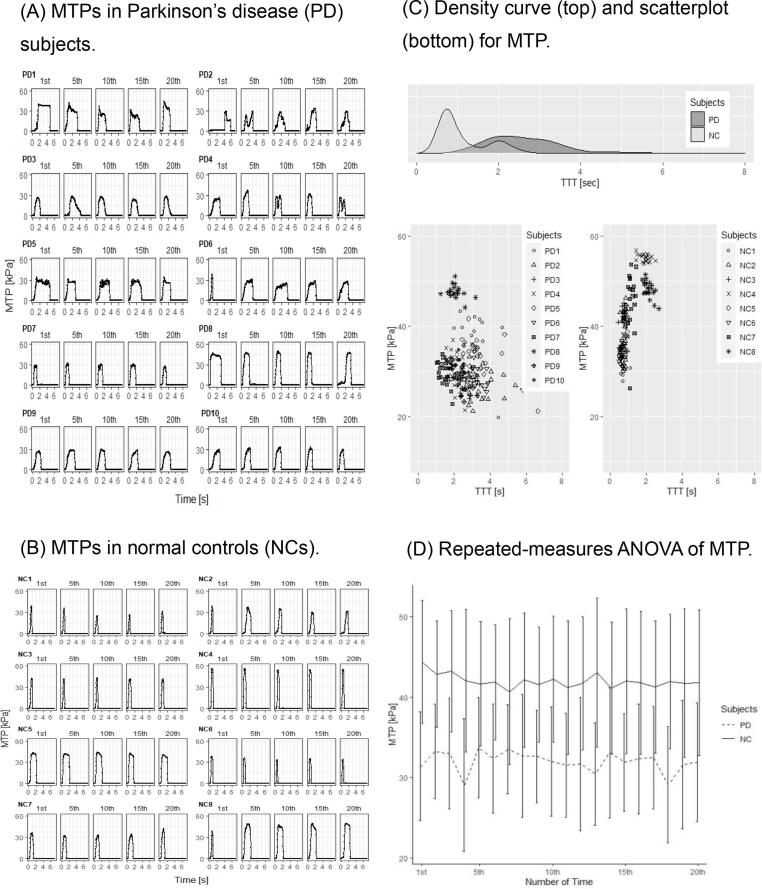
Schematic representation of maximum tongue pressure (MTP). The temporal transition of MTP was similar between some normal controls (NCs) and Parkinson’s disease (PD) subjects, while in other PD subjects (PD #1, #2, #3, #4, #5, #6, and #8) it showed multiple peaks and an irregular, inconsistent pattern that was considerably different from that of NCs (A). In contrast, all NCs showed a consistently monophasic and smooth pattern of MTP generation and temporal transition (B). MTPs were mainly distributed between 20 and 40 kPa, with a total transition time of the tongue pressure (TTT) of 1.0–4.0 s, in PD subjects. MTPs were mainly distributed between 30 and 50 kPa, with a TTT of 0.5–1.5 s, in NCs (C). Neither within- nor between-subject effects related to MTPs were statistically significant in either group (D).

As shown in Fig. 1 A, there were irregular monophasic and polyphasic tongue pressure peaks in 7 of 10 PD subjects, and the type of temporal transition of MTP differed among measurements. The highest tongue pressure was defined as the MTP for PD subjects who showed polyphasic temporal transitions in MTP. In the other PD subjects, the temporal transition of MTP was similar to that of NCs. The tongue pressure in NC subjects reached the MTP more rapidly after tongue pressure onset than in PD subjects, and then quickly returned to baseline; it exhibited a smooth, monophasic pattern (Fig. 1B). The MTP temporal transition was consistent among all NC subjects. The temporal transitions of the other MTPs were similar between PD subjects and NCs (data not shown).

As shown in Fig. 1C, the density curves of MTP for TTT were biphasic in NCs and monophasic in PD subjects.

### 3.2 Internal consistency of MTP and temporal transitions

As shown in Table 1, MTP decreased, and both Ti-Tm and TTT were delayed, in PD subjects compared with NCs (p < 0.05), while there was no significant difference in AUC values between PD subjects and NCs.

There was no statistically significant group difference in MTP according to the mixed-effect ANOVA. Repeated-measures ANOVA, used to assess the internal consistency of MTP, revealed no significant decrement of MTP over time in either group (F = 1.24p = 0.2272; Fig. 1D). Therefore, the between- and within-subjects effects related to MTP were not statistically significant in either group.

## 4 Discussion

Previous studies have shown that MTP is decreased in PD, along with skeletal muscle in the limbs and trunk [2]. Furthermore, the anterior half of the tongue, including the tip, plays an important role in holding and transferring the bolus, serving as an anchor point that provides a base for tongue movement [9]. As shown in Fig. 1A and 1B, MTP generation and temporal transition in PD subjects varied, showing similarity to NCs in some cases and differences in others.

Unlike NCs, the temporal transition of MTP generation revealed by repeated measurements was inconsistent even within the same PD subject. Furthermore, the scatterplot in Fig. 1C shows that it took longer to achieve MTP, which was also distributed over a wider time range, in PD subjects than NCs. Overall, the diversity of the temporal transition of MTP was greater in PD subjects.

Considerable effort is required to repeat MTP 20 times, even with a fixed 10-second interval. However, there was no significant difference in AUC values between the PD subjects and NCs, although the mean MTP was lower, and Ti-Tm and TTT were both delayed, in the former group (Table 1). The decrease in MTP seen in PD subjects may not depend on the tongue pressure in the anterior half of the tongue. If tongue pressure is interpreted as force, the AUC can be expressed as a change in momentum by obtaining an impulse as the area under the force–time curve. However, given that the tongue pressure in the present study was limited to the anterior tongue, including the tip of the tongue, the tongue pressure amount (rather than the momentum of the entire tongue) was obtained as the AUC.

Contrary to the diverse and inconsistent MTP temporal transitions seen in PD subjects, the within- and between-subject effects related to the repeated measurements of MTP were not statistically significant in either group. There was no significant group difference in the AUC values, and the decrease in MTP seen in PD subjects subsequent　to the first measurement was not attributed to peripheral fatigue (i.e., muscle fatigue). However, it should be noted that we repeatedly measured voluntary movements (with a short interval) rather than using a task involving sustained maximum muscle contraction for the assessment of muscle fatigue. In the present study, PD subjects had lower MTP from the first measurement compared with NC subjects, and no significant gradual decrease was observed in subsequent repeated measurements. There was also no gradual decrease in MTP in repeated measurements over time in the NC subjects. Although peripheral fatigue, such as muscle wasting, may taper off with repeated measurements over time, our results did not show a gradual decrease of MTP over time. We therefore believe that our results were not affected by peripheral fatigue.

The reason for the diverse and inconsistent temporal transition of MTP seen in our PD subjects is not clear, and nor is its relationship to the basal ganglia abnormalities that characterize PD. However, the results suggest an inability to stably generate sufficient force [10], which may be associated with bradykinesia [11]. No sequence effect related to bradykinesia was indicated by the repeated measurements method performed in this study, which may be attributable to the use of a 10-second interval rather than requiring the participants to perform continuous repetitive movements (Fig. 1D).

In a study that used functional magnetic resonance imaging to determine whether the basal ganglia show increased activity in response to “pinch grip contractions,” which involve an isometric push force of increasing amplitude, the globus pallidus internus and subthalamic nucleus showed increased signal strength, indicating that these structures modulate force output (i.e., are involved in force scaling) [12]. Therefore, tongue bradykinesia may have played a role in the diverse MTP temporal transitions observed in our PD subjects. However, further studies are needed to accumulate PD subjects and evaluate both MTP transition and bradykinesia severity.

In summary, repeated measurements showed that the MTP decreased significantly over time in the PD subjects compared with that in NCs, and diverse MTP temporal transitions were seen even within the same PD subjects. The results of this study suggest that MTP measurements may shed light on the pathophysiological mechanisms of PD dysphagia and bulbar dysfunction (as reflected in voice impairment, for example), irrespective of disease severity.

## 5 Limitations and future directions

Because this was a pilot study, more participants are needed to fully investigate motor and non-motor symptoms that may be related to decreased MTP in PD patients. In addition, the potential involvement of fatigue and frontal executive function in lowered MTP was not examined in this study. Nevertheless, diversity in MTP was seen in the PD group, even within the same subjects, suggesting the importance of individual-level analyses of MTP in PD populations. In a previous study, subclinical aspiration was observed in > 30 % of healthy elderly subjects, and the aspiration group had significantly lower isometric maximum anterior and posterior tongue pressures than the non-aspirated group [6]. The role of decreased MTP as a risk factor for aspiration pneumonia should be investigated in future, along with its association with both dysphagia and dysphasia. It will also be important to further develop the present study by examining scores of the Montreal Cognitive Assessment (or similar cognitive assessments) to evaluate any differences in cognitive function between controls and PD patients that may affect repeated measures of MTP over time.

## CRediT authorship contribution statement

**Sachi Hayasaka:** Conceptualization, Investigation, Writing – original draft, Visualization. **Kozo Hatori:** Conceptualization, Methodology, Writing – review & editing. **Shuko Nojiri:** Conceptualization, Formal analysis. **Taku Hatano:** Conceptualization, Writing – review & editing. **Takao Urabe:** Conceptualization, Resources. **Akito Hayashi:** Conceptualization, Supervision. **Nobutaka Hattori:** Conceptualization, Supervision. **Toshiyuki Fujiwara:** Conceptualization, Project administration.

## Declaration of competing interest

The authors declare that they have no known competing financial interests or personal relationships that could have appeared to influence the work reported in this paper.
