# Supplementary material for: Novel characteristics of the temporal transition to maximum tongue pressure in Parkinson’s disease: A pilot study
Source: Clin Park Relat Disord. 2024 Feb 25;10:100244. doi: 10.1016/j.prdoa.2024.100244 (PMC10909619; doi:10.1016/j.prdoa.2024.100244)
Supplement: Supplementary data 1 [file mmc1.docx]

**A****ppendix A. Supplementary data.**

**Schematic representation of MTP and parameters of its temporal transition.**


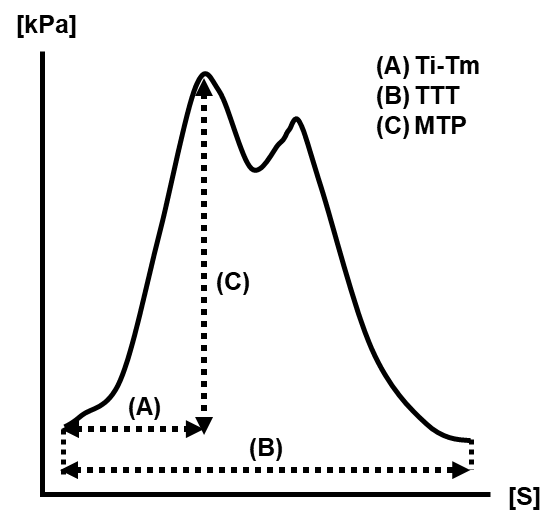


**Ti_Tm, time from the start of tongue pressure generation (Ti) to MTP; TTT, total transition time of the tongue pressure (time to return to baseline); MTP, maximum tongue pressure; kPa,** [**kilopascal**](https://www.weblio.jp/content/kilopascal)**; s, second.**
